# Scoring relevancy of features based on combinatorial analysis of Lasso with application to lymphoma diagnosis

**DOI:** 10.1186/1471-2164-14-S1-S14

**Published:** 2013-01-21

**Authors:** Habil Zare, Gholamreza Haffari, Arvind Gupta, Ryan R Brinkman

**Affiliations:** 1 Department of Genome Sciences, University of Washington, Seattle, Washington, USA; 2 Department of Computer Science, University of British Columbia, Vancouver, BC, Canada; 3 Faculty of Information Technology, Monash University, VIC, Australia; 4 Medical Genetics, University of British Columbia, Vancouver, BC, Canada

## Abstract

One challenge in applying bioinformatic tools to clinical or biological data is high number of features that might be provided to the learning algorithm without any prior knowledge on which ones should be used. In such applications, the number of features can drastically exceed the number of training instances which is often limited by the number of available samples for the study. The Lasso is one of many regularization methods that have been developed to prevent overfitting and improve prediction performance in high-dimensional settings. In this paper, we propose a novel algorithm for feature selection based on the Lasso and our hypothesis is that defining a scoring scheme that measures the "quality" of each feature can provide a more robust feature selection method. Our approach is to generate several samples from the training data by bootstrapping, determine the best relevance-ordering of the features for each sample, and finally combine these relevance-orderings to select highly relevant features. In addition to the theoretical analysis of our feature scoring scheme, we provided empirical evaluations on six real datasets from different fields to confirm the superiority of our method in exploratory data analysis and prediction performance. For example, we applied FeaLect, our feature scoring algorithm, to a lymphoma dataset, and according to a human expert, our method led to selecting more meaningful features than those commonly used in the clinics. This case study built a basis for discovering interesting new criteria for lymphoma diagnosis. Furthermore, to facilitate the use of our algorithm in other applications, the source code that implements our algorithm was released as FeaLect, a documented R package in CRAN.

## Introduction

To build a robust classifier, the number of training instances is usually required to be more than the number of features. In many real life applications such as bioinformatics, natural language processing, and computer vision, a high number of features might be provided to the learning algorithm without any prior knowledge about which ones should be used. Therefore, the number of features can drastically exceed the number of training instances and the model is subject to overfit the training data. Many regularization methods have been developed to prevent overfitting and to improve the generalization error bound of the predictor in this learning situation.

Most notably, Lasso [1] is an *ℓ*_1_-regularization technique for linear regression which has attracted much attention in machine learning and statistics. The same approach is useful in classification because any binary classification problem can be reduced to a regression problem by treating the class labels as real numbers, and consider the sign of the model prediction as the class label. The features selected by the Lasso depends on the regularization parameter, and the set of solutions for all values of this free parameter is provided by regularization path [2]. Although efficient algorithms exist for recovering the whole regularization path for the Lasso [3], finding a subset of highly *relevant *features which leads to a robust predictor is a prominent research question.

In this paper, we propose a novel algorithm for feature selection based on the Lasso and our hypothesis is that defining a scoring scheme that measures the "quality" of each feature can provide a more robust feature selection method. Our approach is to generate several samples from the training data by bootstrapping, determine the best relevance-ordering of the features for each sample, and finally combine these relevance-orderings to select highly relevant features. In addition to the theoretical analysis of our feature scoring scheme, we provided empirical evaluations using a real-life lymphoma dataset as well as several UCI datasets, which confirms the superiority of our method in exploratory data analysis and prediction performance.

### Background and previous work

Lasso is an *ℓ*_1_-regularization technique for least-square linear regression:

(1) $$L:=\overset{n}{\underset{i=1}{\sum }}\frac{1}{2n}{∥y_{i}-w^{T}\cdot \;{\text{x}}_{i}∥}_{2}^{2}+\;\lambda {∥w∥}_{1}$$

where the response random variable *Y *∈ ℝ is dependent on a *d*-dimensional covariate *X *∈ ℝ*^d^*, and the training data $D={\left\{\left(x_{i},y_{i}\right)\right\}}_{i=1}^{n}$ is independently and identically sampled from a fixed joint distribution *P_XY_*. It is well known that the *ℓ*_1_-regularization term shrinks many components of the solution to zero, and thus performs feature selection [4]. There has been also some variants, such as elastic nets [5], to select highly-correlated predictive features. The number of selected features in eqn (1) is controlled by the regularization parameter λ.

A common practice is to find the best value for λ by cross-validation to maximize the prediction accuracy. Having found the best value for the regularization parameter, the features are selected based on the non-zero components of the global and unique minimizer of the training objective in equation (1). However, recent research on the *consistency *of the Lasso [4,6-10] shows that a fixed value of λ for all *n *will not result in a *consistent *estimate for the parameter vector [7]. Now, the question is what would be a proper value for λ as a function of *n *with a theoretical basis?

Various decaying schemes of the regularization parameter were studied [4,7,8,11] and it is shown that under specific settings, Lasso selects the *relevant *features with probability one and the *irrelevant *features with a positive probability less than one, provided that the number of training instances tends to infinity. To do a better feature selection, note that each run of the cross-validation gives the value of the regularization parameter λ *and *the corresponding selected-features. If several samples were available from the underlying data distribution, irrelevant features could be removed by simply *intersecting *the set of selected features for each sample. The idea in [7] is to provide such datasets by resampling with replacement from the given training dataset using the *bootstrap *method [12]. This approach leads to Bolasso algorithm for feature selection that is theoretically motivated by the proposition 1.

**Proposition 1**. [7]*Suppose P_XY _satisfies some mild assumptions and let $\lambda =\mu_{0}n^{-\;\frac{1}{2}}$ for a fixed constant μ*_0 _*>*0. *Let ***J ***represents the index of the ***true ***relevant features, and Ĵ  denote the index of relevant features found by Bolasso. Then, the probability that Bolasso does not select the correct model is upper-bounded by:*

$$\text{Pr}\left(Ĵ\neq J\right)\leq mA_{1}e^{-A_{2}n}+A_{3}\frac{\text{log}n}{n^{\frac{1}{2}}}+A_{4}\frac{\text{log}m}{m},$$

*where m >*1 *is the number of bootstrap samples, and all A_i _s are positive constants*.

Now, if we send *m *to infinity slower than $e^{A_{2}n}$, then with probability tending to one Bolasso will select **J**, exactly the relevant features. The proposition 1 guarantees the performance of Bolasso only asymptoticly, i.e. when *n *→ ∞. However, in real applications where the number of training samples is often limited, the probability of selecting relevant features can be significantly less than 1. One of the main goals of our proposed framework in this paper is to address this problem by scoring the features.

Previous studies have shown that there is room for improving Bolasso [7,8]. For example, while on synthetic data it outperforms similar methods such as ridge regression, Lasso, and bagging of Lasso estimates [13], Bolasso is sometimes too strict on real data because it requires the relevant features to be selected in all bootstrap runs. Bolasso-S, a soft version of Bolasso, performs better in practice because it relaxes this condition and selects a feature if it is chosen in at least a user-defined fraction of the bootstrap replicates (a threshold of 90% is considered to be enough). Bolasso-S is more flexible and thus, more appropriate for the practical models that are not extremely sparse [8].

### Our contributions

In this paper, we develop FeaLect algorithm that is softer than Bolasso in the following three directions:

• For each bootstrap sample, Bolasso considers only one model that minimizes the training objective L  in eqn (1), whereas we include information provided by the whole regularization path,

• Instead of making a binary decision of inclusion or exclusion, we compute a score value for each feature that can help the user to select the more relevant ones,

• While Bolasso-S relies on a threshold, our theoretical study of the behaviour of irrelevant features leads to an analytical criterion for feature selection without using any pre-defined parameter.

We compared the performance of Bolasso, FeaLect, and Lars algorithms for feature selection on six real datasets in a systematic manner. The source code that implements our algorithm was released as FeaLect, a documented R package in CRAN.

### Feature scoring and mathematical analysis

In this section, we describe our novel algorithm that scores the features based on their performance on samples obtained by bootstapping. Afterwards, we present the mathematical analysis of our algorithm which builds the theoretical basis for its proposed automatic thresholding in feature selection.

### The FeaLect algorithm

**Figure 1:**
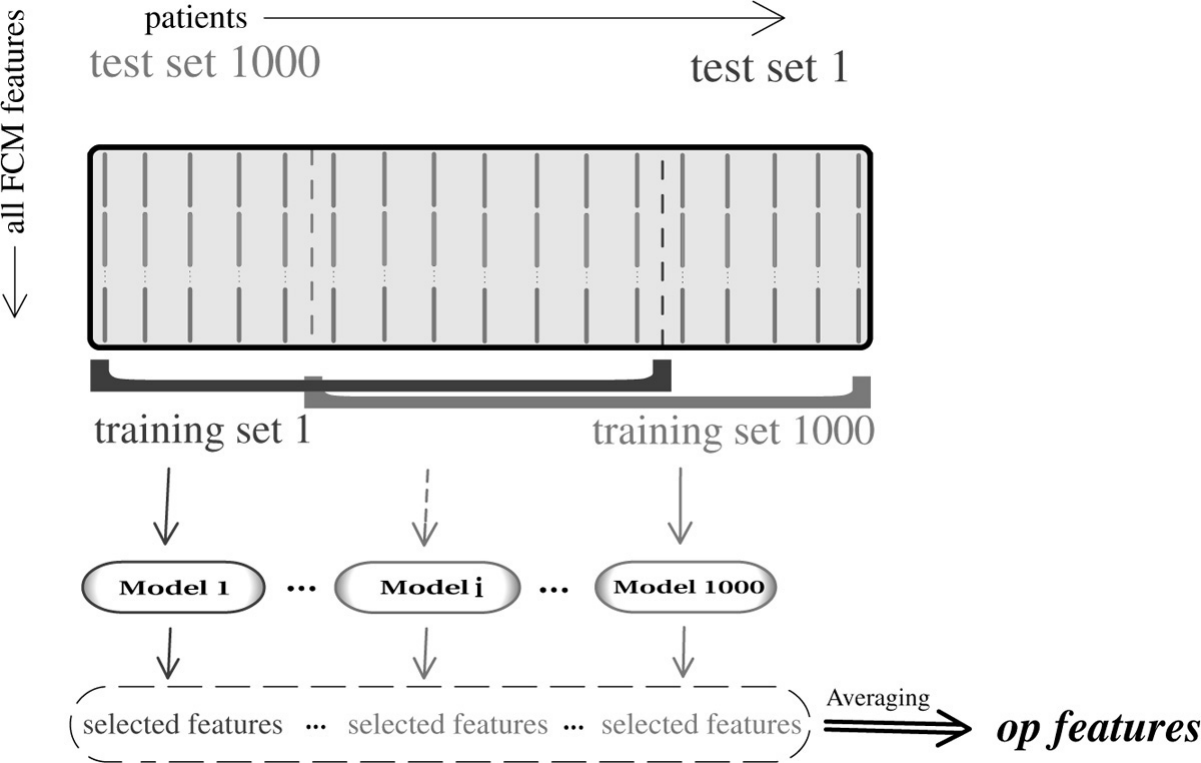
**Overview of bootstraping performed by FeaLect**. A row and a column of the gray data matrix correspond to a feature and a case, accordingly. 1000 models are trained, each fitted to a random subset that contains $\frac{3}{4}$ of cases using Lasso technique [1]. Without any assumption from a-priori knowledge, all features are included for training the models. Then the selected features are scored by computing an average vote (eq. 3) to select the most predictive ones.

Our feature selection algorithm is outlined in Figure 1 and described in Algorithm 1. Let *B *be a random sample with size *γn *generated by choosing from the given training data *D without *replacement, where *n *= |*D*| and *γ *∈ (0, 1) is a parameter that controls the size of sample sets. Using a training set *B*, we apply the Lars algorithm to recover the whole regularization path efficiently [3]. Let $F_{k}^{B}$ be the set of selected features by the Lasso when λ allows exactly *k *features to be selected. The number of selected features is decreasing in λ and we have:

$$\emptyset =F_{0}^{B}\subset \ldots F_{k}^{B}\subset F_{k+1}^{B}\subset \cdots \subset F_{d}^{B}=F.$$

For each feature *f*, we define a scoring scheme depending on whether or not it is selected in $F_{k}^{B}$:

(2) $$S_{k}^{B}\left(f\right):=\left\{\begin{array}{cc} \frac{1}{k} & \text{if}\;f\in F_{k}^{B}\\ 0 & \text{otherwise} \end{array}\right)$$

**Figure 2:**
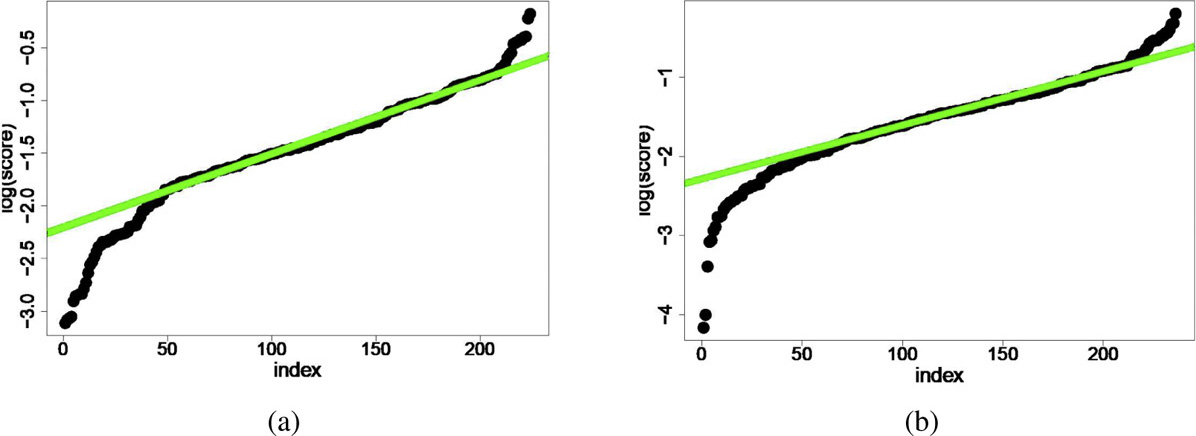
**Total feature scores in the log-scale**. The middle-part of the curves is linear and represents scores of the irrelevant features (see section). The scores in (a) and (b) diagrams are computed by 1000 and 5000 samples, respectively. The low variance between diagrams indicates fast convergence and stability of score definition. Data is from lymphoma dataset.

The above randomized procedure is repeated several times for various random subsets *B *to compute the *average *score of *f *when exactly *k *features are selected, i.e. $E_{B}\left[S_{k}^{B}\left(f\right)\right]$ is estimated empirically. According to our experiments, the convergence rate to the expected score is fast and there is no significant difference between the average scores computed by 100 or 1000 samples (Figure 2). The total score for each feature is then defined as the sum of average scores:

(3) $$S\left(f\right):=\underset{k}{\sum }E_{B}\left[S_{k}^{B}\left(f\right)\right]$$

**Algorithm 1 **Feature Scoring

**1:   for **t = 1 to *m ***do**

**2:**      Sample (without replacement) a random subset *B *⊂ *D *with size *γ*|*D*|

**3:**      Run Lars on *B *to obtain $F_{1}^{B}$, ...,$F_{d}^{B}$

**4:**      Compute $S_{1}^{B}$, ...,$S_{d}^{B}$ using eqn (2)

**5:**      **for ***k *∈ {1, ..., *d*} **do**

**6:**         Update the feature scores for all feature $f:S(f\leftarrow S\left(f\right)+S_{k}^{B}\left(f\right)/m$

**7:**      **end for**

8:   end for

**9:**   Fit a 3-segment spline (*g*_1_(.), *g*_2_(.), *g*_3_(.)) on log-scale feature score curve (see the text for more information)

**10:   return **features corresponding to *g*_3 _as informative features

Before describing the rest of the algorithm, let us have a look at the feature scores for our lymphoma classification problem (The task and data set is described in details in the Experiment section). Figure 2 depict the total score of features in log-scale, where features are sorted according to their increasing total scores. The feature score curve is almost linear in the middle and bending at both ends. We hypothesize that features with a "very high score" in the top non-linear and bending part of the curve are good candidates for informative features. Furthermore, the linear middle-part of the curve consists of features that are responsible for the model to get overfitted and therefore we call them *irrelevant *features. In the next section, a formal definition will be provided to clarify this intuitive idea and we show how this insight can be very helpful in identifying informative features.

The final step of our feature selection algorithm is to fit a 3-segment spline model to the feature score curve: the first quadratic lower-part captures the low score features, the linear middle-part captures irrelevant features, and the last quadratic upper-part captures high-score informative features. As discussed below, the middle linear-part provides an analytic threshold for the score of relevant features: The features with score above this threshold are reported as informative features which can be used for training the final predictor and/or explanatory data analysis.

### The analysis

The aim of this analysis is to provide a mathematical explanation for the linearity of the middle part of the scoring function (Figure 2), and also a justification for why the features corresponding to this part can be excluded. We first present a probabilistic interpretation of the feature scores. and then we provide a precise definition of an irrelevant feature. Our definition formalizes the fact that such a feature is selected by the Lasso if and only if a particular fixed finite subset *U *of instances is included in the random training set, whereas a relevant feature should be selected for almost any general *U*. We estimate the probability that a random sample *B *⊂ *D *contains *U *as *n *grows to infinity. Finally, we show that asymptotically, the log of the scores for irrelevant features is linear in |*U*|. This explains the linearity of the middle part of the feature score curve in Figure 2.

**Proposition 2**. *Suppose *Pr (**f **= *f_i_*) *is the probability of selecting a feature f_i _by the Lasso in some stage of our feature selection method in Algorithm 1. Then, the probability distribution of the random variable ***f ***is given by:*

$$\text{Pr}\left(f=f_{i}\right)=\frac{1}{d}S\left(f_{i}\right)$$

*Proof*. By conditional probability:

$$\begin{array}{c} \text{Pr}\left(f=f_{i}\right)\;=\underset{B}{\sum }\;\;\text{Pr}\left(f=f_{i}|B\right)\text{Pr}\left(B\right)\\ \;\;\;\;\;=\underset{B}{\sum }\overset{d}{\underset{k=1}{\sum }}\;\left(\text{Pr},(,f=f_{i}|f\in F_{k}^{B}\right)\\ \;\;\;\;\;\;\;\;\;\;\left(\text{Pr}\left(f\in F_{k}^{B}\right)\text{Pr}\left(B\right)\right)\\ \;\;\;\;\;=\underset{B}{\sum }\overset{d}{\underset{k=1}{\sum }}\;S_{k}^{B}\left(f_{i}\right)\text{Pr}\left(f\in F_{k}^{B}\right)\text{Pr}\left(B\right) \end{array}$$

Since we have not imposed any prior assumption, we put a uniform distribution on $\text{Pr}\left(f\in F_{k}^{B}\right)$ to get:

$$\begin{array}{c} \text{Pr}\left(f=f_{i}\right)\;=\frac{1}{d}\underset{B}{\sum }\underset{k}{\sum }S_{k}^{B}\left(f_{i}\right)\text{Pr}\left(B\right)\\ \;\;\;\;\;=\frac{1}{d}E_{B}\left(\underset{k}{\sum }S_{k}^{B}\left(f_{i}\right)\right)\\ \;\;\;\;\;=\frac{1}{d}S\left(f_{i}\right). \end{array}$$

□

The following definition formalizes the idea that irrelevant features depend only on a specific subset of the whole data set.

**Definition 3**. *For any subset of samples U *⊆ *A and any feature f_i_, we say that f_i _over-fits on U if:*

$$\forall k,\forall B:f_{i}\in F_{k}^{B}\Leftrightarrow U\subseteq B$$

In words, *f_i _*is selected in $F_{k}^{B}$ if and only if *B *contains *U*. Next, we derive the probability of including a specific set *U *in a randomly generated sample.

**Lemma 4**. *For any U *⊆ *A, we have:*

$$\underset{n\to \infty }{\text{lim}}\underset{B}{\text{Pr}}\left(U\subseteq B\right)=\gamma^{r}$$

*where r is the number of samples in U and γ is the fraction of samples chosen for a random set B*.

*Proof*. Assuming *B *has *γn *members chosen without replacement, we have:

$$\begin{array}{c} \underset{B}{\text{Pr}}\left(U\subseteq B\right)=\frac{\left({}_{\gamma n-r}^{n-r}\right)}{\left({}_{\gamma n}^{n}\right)}\\ \;\;\;\;\;\;=\frac{\left(n-r\right)!\left(\gamma n\right)!}{n!\left(\gamma n-r\right)!}\\ \;\;\;\;\;\;=\left(\overset{n-r}{\underset{i=1}{\prod }}i\right)\left(\overset{\gamma n}{\underset{i=1}{\prod }}i\right)\left(\overset{n}{\underset{i=1}{\prod }}i^{-1}\right)\left(\overset{\gamma n-r}{\underset{i=1}{\prod }}i^{-1}\right)\\ \;\;\;\;\;\;=\left(\overset{n-r}{\underset{1}{\prod }}i\right)\cdot \left(\overset{\gamma n-r}{\underset{1}{\prod }}i\right)\;\cdot \;\left(\overset{\gamma n}{\underset{\gamma n-r+1}{\prod }}i\right)\times \\ \;\;\;\;\;\;\left(\overset{n-r}{\underset{1}{\prod }}i^{-1}\right)\cdot \left(\overset{n}{\underset{n-r+1}{\prod }}i^{-1}\right)\;\cdot \;\left(\overset{\gamma n-r}{\underset{1}{\prod }}i^{-1}\right)\\ \;\;\;\;\;\;=\left(\overset{\gamma n}{\underset{\gamma n-r+1}{\prod }}i\right)\;\cdot \;\left(\overset{n}{\underset{\gamma n-r+1}{\prod }}i^{-1}\right)\\ \;\;\;\;\;\;=\overset{r-1}{\underset{i=0}{\prod }}\left[\left(\gamma n-i\right){\left(n-i\right)}^{-1}\right]\\ \;\;\;\;\;\;=\gamma^{r}\overset{r-1}{\underset{i=0}{\prod }}\left(\frac{n-\frac{i}{\gamma }}{n-i}\right)\\ \;\;\;\;\;\;=\gamma^{r}\overset{r-1}{\underset{i=0}{\prod }}\left(1+\frac{i\left(1-1/\gamma \right)}{n-i}\right)\\ \;\;\;\;\;\;=\gamma^{r}\left(1+O\left(n^{-1}\right)\right). \end{array}$$

The first line of the above proof relies on the assumption that the members of the random set *B *are chosen without replacement, and the claim derives from the fact that *γ *is a fixed constant. □

The following theorem concludes our argument for the exponential behavior of total score of irrelevant features. It relates the probability of selecting a feature *f_i _*irrelevant on *U *to the probability of including *U *in the sample.

**Theorem 5**. *If a feature f_i _over-fits on a set of samples U with size r, then:*

$$\underset{n\to \infty }{\text{lim}}S\left(f_{i}\right)=d\gamma^{r}.$$

*Proof*. From proposition 2 we have:

$$\begin{array}{c} S\left(f_{i}\right)=d\text{Pr}\left(f=f_{i}\right)\\ \;\;\;=d\underset{B}{\sum }\text{Pr}\left(f_{i}\in F^{B}\right)\text{Pr}\left(B\right)\sum \text{Pr}\\ \;\;\;=d\underset{B}{\text{Pr}}\left(U\subseteq B\right)\\ \;\;\;=d\left(\gamma^{r}+O\left(n^{-1}\right)\right). \end{array}$$

The last equation was proved in lemma 4, and the one before that from definition 3. □

Although we presented the above arguments for the Lasso, it also should work for any other feature selection algorithm which exhibits linearity in its feature score curve. That is, features corresponding to the linear part of the scoring curve are indeed the irrelevant features for that algorithm, and therefor, the features on non-linear upper-part should be considered as informative ones. Obviously the features on the non-linear lower-part are not interesting for the any prediction task because their scores are even less than the irrelevant features. We speculate that these features do not present a linear behavior because not only they are not relevant to the outcome, but also they are not associated with any particular set *U*, meaning they are not even included in an over-fitted model. A follow-up study may investigate this hypothesis further.

## Experiment with real data

We applied FeaLect on several datasets to test the performance of our feature selection algorithm in real life conditions.

### Lymphoma

Lymphoma is a cancer that begins in the lymphatic cells of the immune system, and is presented as a solid tumor of lymphoid cells [14]. Just as cancer represents many different diseases, lymphoma represents many different cancers of lymphocytes [15]. We applied our algorithm for automatic diagnosis of lymphoma types based on flow cytometry (FCM) data [16]. Usually 15-30 markers are used for each patient, where each marker distinguishes a particular cell type based on its protein content. We analyzed flow cytometry data of 85 lymphoma patients who had been diagnosed at BC Cancer Agency, Vancouver, Canada between 2004-2007. The patients were grouped into four top-level disease subgroups and the goal was to build a classifier that could diagnosis 20 test patients based on their FCM data. For each group, we trained a classifier to distinguish that group versus the others. These four classifiers were then combined to provide the top-level diagnosis.

#### Data preparation and feature extraction

The blood sample of each patient was divided into 7 portions, and each portion is examined in a different tube by the cytometer. Each tube gives 5 dimensional data of 20,000-70,000 blood cells. In the first analysis step, we used a spectral clustering approach to cluster the cells in each tube into cell populations. It was not possible to directly apply classical spectral clustering [17-20] to the lymphoma data because it involved computing eigenvectors of a big *n*-by-*n *matrix where *n *ranges from 20,000 to 70,000. Instead, we have made use of SamSPECTRAL that is our enhanced spectral clustering method capable of analyzing large amount of data in a reasonable amount of time; it has also a good memory footprints [21].

SamSPECTRAL performs a specific sampling stage called *faithful sampling *to reduce the size of data for spectral clustering. Our data reduction scheme is designed to preserve density information and can be briefly stated as follows:

1. Set all points to be unregistered and assume the parameter *h *is adjusted appropriately.

2. Pick a random unregistered point *p *(the representative of a community) and find all unregistered data points within distance *h *from *p*.

3. Put all of these points in a set called community *p*, and label them as registered.

4. Repeat the above two steps until no unregistered points are left.

After the above steps, the similarity between the communities is defined by summing up similarities between their members, and the resulting similarity matrix is passed to a classical spectral clustering algorithm. Because this matrix is much smaller than the original similarity matrix (3000-by-3000 instead of 20,000-by-20,000 in our experiments), its eigenvectors can be efficiently computed in reasonable time.

Each cluster computed by SamSPECTRAL was regarded as a "cell population" that could potentially have information about the lymphoma type. Without imposing any *a priori *knowledge on the importance of any population, we considered their sizes and their means in all dimensions as features. In total, 276 features were obtained and ignoring those with very low variance, 224 were kept for feature selection and classification.

#### Feature selection and classification

Since the number of features was considerably larger than the number of training samples (*p *= 224, *n *= 85), a careful feature selection scheme was needed. To reduce the computation time required, we imposed a pre-defined upper bound 60 on the number of features based on *a priori *knowledge from the biology. We initially applied *ℓ*_1 _-regularization technique, and it was not by its own enough to prevent overfitting. Reducing the regularization parameter did not improve the results as we observed that some of the features that were known to be biologically and clinically interesting were ignored. We also applied Bolasso [7] to select relevant features. For most bootstrap samples of our data, the global error defined by equation (1) was minimized when only a few (less than 4) features were selected. Because the intersection of selected features from several (more than 10) samples was empty, Bolasso could not result in appropriate feature selection.

Next, we applied our feature selection algorithm. In our experiment, we set $\gamma =\frac{3}{4}$ to be the fraction of instances used in each iteration for training. Figures 2(a) and 2(b) depict the resulting feature scores for follicular lymphoma type after *m *= 1000 random runs. The log-scale plot consisted of a linear part that confirmed our hypothesis experimentally. Similarly, the plots for other types of lymphoma had also linear parts. Furthermore, we re-ran the experiments with *m *= 5000 random samples and the results did not vary significantly indicating a fast convergence rate for the feature scores.

To select the informative features, we fitted a 3-segment spline model to each curve. The features corresponding to the middle linear segment were considered as irrelevant ones, and ignored for the rest of analysis. Features with score higher than score of these irrelevant features were selected as informative features. We observed that unlike the pure Lasso, all features that were known to be biologically and clinically interesting were selected by our approach. Prediction accuracy was improved confirming the efficiency of our feature selection method. We used our selected features to build a linear classifier that had precision, recall and F-measure 98%, 94% and 96%, respectively while the best result we obtained with the pure Lasso was 93%, 82% and 87%, respectively.

For further evaluation in a data explarotary setting, we interrogated the *selected features *together with our clinical collaborators for novel biomarker discovery. A task which would be challenging otherwise, due to large number of features and huge amount of clinical work required to evaluate each individual feature. We narrowed down our attention to those features which were relevant to lymphoma types based on our feature selection algorithm, but were not previously reported to be biologically relevant. This approach resulted in the discovery of interesting new criteria for lymphoma diagnosis that have clinical applications in practice [22].

### Additional real datasets

In addition to our lymphoma flow cytometry data, we validated the performance of FeaLect on five other datasets including the well-known colon gene expression (Table 1). Colon dataset contains expression of 2000 genes in 22 normal and 40 colon cancer tissues [23] and it is a benchmark for gene expression analysis [24]. All additional four datasets are from UCI (University of California, Irvine) Machine Learning Repository [25]. Arcene contains mass-spectrometric data for cancer and normal cases [26], variables of SECOM were collected from sensors and process measurements in complex modern semi-conductors with the goal of enhancing current business improvement techniques [25]. We used a version of SECOM dataset balanced by randomly selecting equal number of positive and negative samples. The learning task for Connectionist dataset is to train a network to discriminate between sonar signals bounced off a metal cylinder and those bounced off a roughly cylindrical rock [27]. ISOLET is a natural language processing dataset generated from speech of 150 subjects with the goal of identifying which letter-name was spoken [28]. In the current study, we only considered letters A and B as positive and negative samples and excluded the rest of samples.

**Table 1:** Comparsion of area under the ROC curve between FeaLect, lars, and Bolasso on six different datasets.

Dataset	Total samples	# of features	20 training samples			40 training samples			Reference
					
			Bolasso	lars	FeaLect	Bolasso	lars	FeaLect	
Lymphoma	258	505	0.62	0.81	0.84	0.67	0.87	0.88	current
Colon	62	2000	0.50	0.57	0.65	0.47	0.64	0.75	[23]
Arcene	100	10000	0.51	0.59	0.64	0.50	0.66	0.72	[26] (UCI)
SECOM	208	590	0.51	0.57	0.61	0.52	0.61	0.64	[25] (UCI)
Connectionist	208	60	0.63	0.76	0.78	0.67	0.78	0.79	[27] (UCI)
ISOLET	479	617	0.90	0.99	1.00	0.91	1.00	1.00	[28] (UCI)

**Figure 3:**
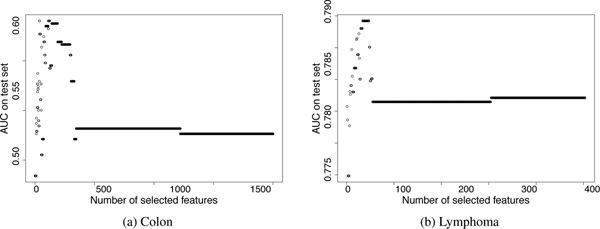
**Variation of area under the ROC curve when different number of features are used**. The features are sorted by applying FeaLect on 20 random training samples. Then, the training samples and the highly scored features are considered to build linear classifiers by lars. The best AUC is reported by testing on a set of validating samples disjoint from the training set. For both lymphoma and colon datasets, the performance of the optimum classifier decreases if all features are provided to lars. This observation practically shows the advantage of using a limited number of highly scored features over pure lars.

**Figure 4:**
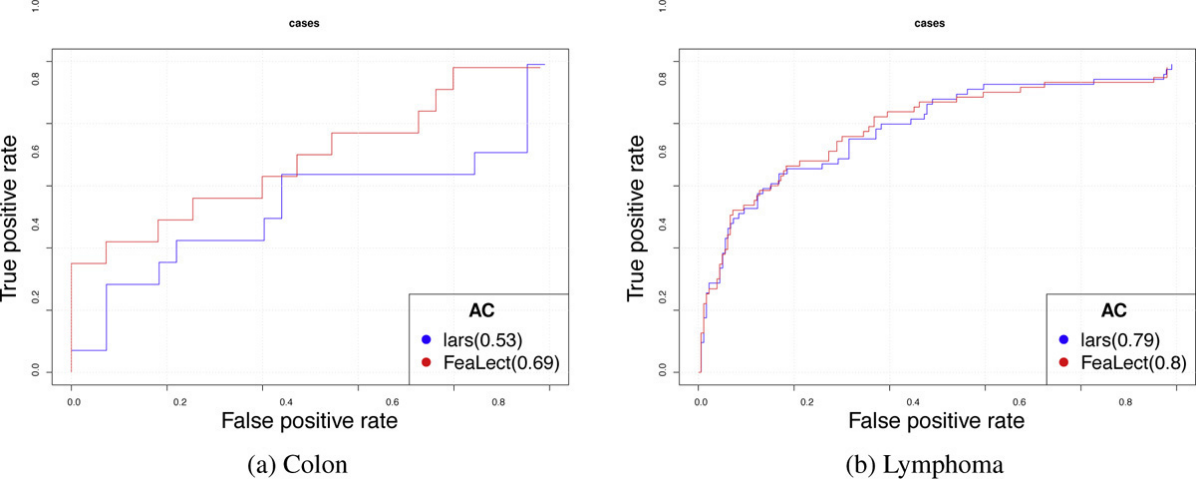
**Comparing ROC curves between FeaLect and lars**. The blue curve represents the ROC curve of the best Lasso model trained on 20 random samples using all available features, and the red curve shows the performance of the best Lasso model when only 61 and 36 top features are provided from colon and lymphoma datasets respectively. While FeaLect always performs better than pure lars, the difference is more significant for colon dataset than lymphoma dataset.

**Figure 5:**
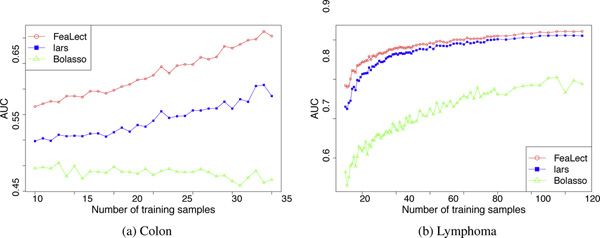
**Improvements in the area under the ROC curves by increasing the number of training samples**. Except for Bolasso on colon dataset, the average performance increases as more training samples are provided. While FeaLect and lars converge to a common asymptotic performance on lymphoma dataset, FeaLect is consistently superior to pure lars on colon dataset because the number of training samples is very limited. Table 1 presents similar superiority for other datasets with relatively low instances.

Table 1 compares the performance of Bolasso, pure lars, and FeaLect on the studied datasets. Training samples were selected uniformly at random and area under the ROC curves (AUC) were computed using the rest of samples (Figure 3). For each dataset, we repeated this procedure 100 times and reported the average AUC to avoid any dependency on the random selection of train-test sets. Both FeaLect and lars always outperformed Bolasso. When only 20 random training samples were provided, FeaLect provides significantly better than pure lars except ISOLET dataset. The number of samples in ISOLET dataset is more than other datasets, enough such that both methods performed well. The superiority of FeaLect over lars decreases as the number of training samples increases from 20 to 40, except for Colon and Arcene datasets for which FeaLect is still better than lars by .09 and 0.6, accordingly. Interestingly, these two datasets are the ones with 2000 and 10000 features that are considerably higher dimensional than other datasets. This observation reassures that FeaLect is advantageous over lars in high-dimensional settings and their performance converges as "adequate" number of samples are provided (Figures 4 and 5).

## Conclusion

We have presented FeaLect, a novel feature selection algorithm, based on Lasso (Figure 1). The idea of FeaLect is to combine the selected feature sets to score the features according to their relevancy and prediction power. An advantage of FeaLect compared to many other feature selection methods is to provide a ranking for features relevance, which can be leveraged in better prediction models and/or exploratory data analysis. We reported a cancer classification problem (lymphoma diagnosis) for which distinguishing the most relevant features is of great interest from the biological and clinical point of view. FeaLect has led to the discovery of novel biomarkers for this disease to help clinicians in lymphoma subtype diagnosis [22]. The log-scale score curve can be studied in more detail and explaining its behavior in the non-linear parts is potentially a source of insight. Shedding more light on the Lasso performance by studying feature scores is a possible future direction of this study.

Furthermore, we provided empirical and quantitative evaluations on five other real-world datasets (from different fields) to confirm the superiority of our method, in prediction performance, compared to the baselines.

## Competing interests

The authors declare that they have no competing interests.

## Authors' contributions

AG and RB supervised the project and motivated the study by providing scientific insight. HZ developed the idea of scoring features and performed the experiments. HZ and GH designed the mathematical analysis. RB provided data and computing facilities. All authors read, edited and approved the final manuscript.

## Declarations

The research and publication costs for this article were funded by NIH grants 1R01EB008400 and 1R01EB005034, the Michael Smith Foundation for Health Research, the National Science and Engineering Research Council and the MITACS Network of Centres of Excellence.

This article has been published as part of *BMC Genomics *Volume 14 Supplement 1, 2013: Selected articles from the Eleventh Asia Pacific Bioinformatics Conference (APBC 2013): Genomics. The full contents of the supplement are available online at http://www.biomedcentral.com/bmcgenomics/supplements/14/S1.
